# Prior Forage Type Influences Ruminal Responses to a Wheat Grain Challenge in Lactating Dairy Cows

**DOI:** 10.3390/ani11113188

**Published:** 2021-11-08

**Authors:** Victoria M. Russo, Brian J. Leury, Emer Kennedy, Murray C. Hannah, Martin J. Auldist, Greg L. Morris, William J. Wales

**Affiliations:** 1 Agriculture Victoria, Ellinbank, VIC 3821, Australia; victoria.russo@agriculture.vic.gov.au (V.M.R.); murray.hannah@agriculture.vic.gov.au (M.C.H.); greg.morris@agriculture.vic.gov.au (G.L.M.); bill.wales@agriculture.vic.gov.au (W.J.W.); 2 Faculty of Veterinary and Agricultural Sciences, The University of Melbourne, Parkville, VIC 3010, Australia; brian.leury@unimelb.edu.au; 3 Centre for Agricultural Innovation, School of Agriculture and Food, Faculty of Veterinary and Agricultural Sciences, The University of Melbourne, Parkville, VIC 3010, Australia; 4 Teagasc, Animal and Grassland Research and Innovation Centre, Moorepark, Fermoy, Co., Cork, Ireland; emer.kennedy@teagasc.ie

**Keywords:** acidosis, adaptation, lucerne hay, perennial ryegrass, ruminal pH

## Abstract

**Simple Summary:**

High-producing dairy cows require more than just pasture to meet the energy demands of milk production. Wheat is an excellent energy source for milk production; however, cows require careful adaptation and monitoring to avoid ruminal upset when large amounts of wheat are introduced. The results of this study show that careful selection of the forage that precedes wheat could allow safer and more aggressive grain introduction strategies to be used in the dairy industry.

**Abstract:**

To increase the dry matter and metabolisable energy intake of cows, dairy farmers often supplement pasture with concentrates and conserved fodder. Feeding large amounts of highly fermentable concentrates to cows can result in metabolic issues, such as ruminal acidosis, and thus safer but more efficient introduction strategies are desirable. We assessed the role that forages play in ruminal, behavioural and production responses to a wheat grain challenge in dairy cows with no previous wheat adaptation. Multiparous lactating Holstein dairy cows (*n* = 16) were fed a forage-only diet of either lucerne (*Medicago sativa*) hay, perennial ryegrass (*Lolium perenne* L.) hay or one of two cultivars of zero-grazing fresh perennial ryegrass herbage (Bealey or Base), for 3 weeks. The forage diet was then supplemented with crushed wheat grain at 8 kg dry matter/cow day^−1^, with no adaptation period. Wheat comprised between 32 and 43% of total dry matter intake. Cows fed hay maintained a higher mean ruminal fluid pH than those fed herbage, on both the forage-only diet (6.43 vs. 6.17) and the forage plus wheat diet (6.03 vs. 5.58). Following supplementation of wheat, cows fed herbage exhibited minimum ruminal fluid pH levels indicative of acute ruminal acidosis, at 5.15 and 5.06 for cultivars Bealey and Base, respectively. Furthermore, for both herbage cultivars, adding wheat resulted in a ruminal fluid pH under 6 for >20 h/day. The ruminal environment of cows fed lucerne hay remained most stable throughout the grain challenge, spending the least amount of time below pH 6.0 (9.0 h/day). Hay created a ruminal environment that was better able to cope with the accumulation of acid as wheat was digested. A combination of increased ruminating time and a slower rate of fermentation, due to higher neutral detergent fiber and lower metabolisable energy concentrations in the hays, is likely responsible for the higher ruminal fluid pH values. Forage plays a critical role in wheat introduction strategies; aggressive adaptation strategies could be implemented when a hay such as lucerne is used as the base forage.

## 1. Introduction

Although most dairy farms in Victoria, Australia, rely on grazed pasture as their main feed source, pasture alone cannot fully meet the nutritional requirements of a high-producing dairy cow [1]. Both dry matter intake (DMI) and metabolizable energy limit milk production on a pasture-only diet [2]. Due to this, grazing dairy cows are typically fed supplementary nutrients, commonly cereal grains or pelleted concentrates offered twice daily in the dairy during milking and, at certain times, conserved fodder fed in the paddock. In Australia, wheat and barley grains are the most commonly used grains and are typically fed at an average rate of 1.6 t/cow year^−1^ [3]. The amount and type of concentrates fed at different stages of lactation can be altered to reflect the nutrients supplied from pasture and the energy requirements of the cows, known as stepped flat rate feeding [4]. A sudden introduction or increase in the amount of starch offered during stepped flat rate feeding can cause dramatic changes to the ruminal environment, including a rapid increase in acid production as a result of fermentation, to which ruminal microbes require time to adapt. If large quantities of concentrates are introduced abruptly to unadapted cows, the ruminal environment may not be able to cope with the increased acid load, leading to metabolic issues such as acute acidosis or sub-acute ruminal acidosis [5]. Therefore, adaptation processes are typically implemented over several weeks with the amount of concentrates being offered, gradually increasing.

A ruminal fluid pH below 6.0 for extended periods of time can severely inhibit fibre digestion [6]; hence, a lower threshold of pH 6.0 is typically used to identify optimal ruminal function. While it is commonly the feeding of concentrates that causes reductions in ruminal fluid pH, the responses in the rumen to different forages are not always equal. For example, Williams et al. [7] reported a ruminal fluid pH consistently below 6.0 when dairy cows were consuming highly digestible fresh Persian clover (*Trifolium resupinatum*) or grazing perennial ryegrass (*Lolium perenne* L.). In contrast, Leddin et al. [8] reported a ruminal fluid pH that remained consistently above 6.0 when lactating dairy cows were consuming a diet of solely perennial ryegrass hay. Furthermore, the rate at which ruminal fluid pH declines can be greater for cows fed legumes compared to cows fed grass [9]. Ruminal responses to increasing amounts of crushed wheat grain also vary depending on forage type [8,10]. Eating behaviour and intake rates vary with forage type, and both impact ruminal fluid pH, mainly through saliva production [11,12]. Introducing or increasing concentrate supplements in a forage-based diet also alters eating behaviour, with both the amount of time spent eating and ruminating decreasing as the proportion of wheat in the diet increases [13].

The process of gradually adapting cows to large amounts of concentrates can come at a cost of production, convenience and efficiency. It is therefore desirable to accelerate the process while still optimizing rumen function and milk production. This experiment investigated the effects of different forages during the abrupt introduction of wheat grain, with the aim of providing some insight into the possibility of using forages for improving concentrate adaptation processes. The hypotheses tested were that (1) the amount of time per day that ruminal fluid pH was below 6.0 would be greater for fresh forages compared to hays; (2) there would be no difference in the time per day that ruminal fluid pH was below 6.0 for the two fresh forages, nor between the two hays; (3) the minimum ruminal fluid pH would be lowest for cows fed fresh cut perennial ryegrass herbage compared to hays; and (4) the minimum ruminal fluid pH would not differ between the two fresh forage treatments, nor between the two hays.

## 2. Materials and Methods

The experiment was conducted at the Agriculture Victoria Research Centre, Ellinbank, Victoria, Australia (38°14′ S, 145°56′ E), in September 2017. All procedures were conducted in accordance with the Australian Code of Practice for the Care and Use of Animals for Scientific Purposes [14]. Approval to proceed was obtained from the Agricultural Research and Extension Animal Ethics Committee, application number 2017-06, and was contingent on having thresholds for minimum ruminal fluid pH for removal of cows (pH 5.0).

Sixteen rumen-fistulated Holstein Friesian dairy cows in their third to ninth lactation were used. While all cows were seasonally calving, a combination of both fresh and carryover cows was used, either having calved between July and October 2016 or 2017 (230 ± 163.1 DIM; mean ± SD). Milking occurred twice daily at ~0600 and 1500 h. Twenty-one days prior to the experiment, concentrates being offered to the cows were gradually reduced, and for the final seven days prior to the experiment, they were fed a forage-only diet. The experiment then ran for 24 days comprising a 3-day covariate period, a 17-day adaptation period and a 4-day measurement period. During the covariate period, all cows grazed perennial ryegrass as a single cohort and received no concentrates. Following the covariate period, four treatments were randomly allocated to cows, such that the treatment groups were balanced for mean ruminal fluid pH (6.4 ± 0.20 pH; mean ± SD), milk yield (milk yield, 27.0 ± 8.63 kg/cow day^−1^), body weight (617 ± 47.1 kg), DIM (230 ± 163.1 DIM) and age (8.1 ± 2.11 years), as recorded during the covariate period.

Each treatment group received one of the following forages: lucerne hay, perennial ryegrass hay, fresh perennial ryegrass cultivar Bealey or fresh perennial ryegrass cultivar Base. During the adaptation period, all cows were moved to individual indoor pens for feeding and were offered their allocated forage ad libitum. Both cultivars of perennial ryegrass were harvested to 5 cm above ground level immediately before being offered to the cows. Cows were not given any concentrates during the adaptation period. In between feeding bouts, cows were returned to a bare paddock with no feed but with free access to water. During the measurement period, forage was offered at a rate of 17 kg DM/cow day^−1^. For the first 2 days of the measurement period, all cows were fed only forage. On days 3 and 4, crushed wheat grain was offered at a rate of 8 kg DM/cow day^−1^, and forage continued to be offered at a rate of 17 kg DM/cow day^−1^. Following each milking, cows were moved to individual stalls and given half their ration in the morning and half in the evening. Wheat was offered first, and within 20 min any grain refusals were removed, and forage was offered. All cows were given 4.5 h to consume their forage and had free access to water during this time.

The experiment was designed with four measurement days. However, due to several cows reaching the designated minimum ruminal fluid pH thresholds (pH 5.0), as required by the presiding animal ethics committee, the experiment was concluded 6 h after the morning feed on day 4. No data collected on day 4 were included in the analyses.

All feed offered and refused was weighed, and a representative sample was collected at each feeding. Part of each sample was then dried at 100 °C for 24 h to determine the DM concentration, which facilitated the calculation of individual DMI. The remainder of the samples were then bulked by feed type or, in the case of refusals, by individual cow and stored at 4 °C. At the completion of the experiment, bulked samples were thoroughly mixed and representative sub-samples were freeze dried and ground to pass through a sieve with mesh apertures of 1 mm. The samples were then analysed for crude protein (CP), acid detergent fibre (ADF), neutral detergent fibre (aNDF), lignin, non-fibre carbohydrates (NFC), starch, crude fat (CF), ash and estimated metabolisable energy (ME) by wet chemistry in a commercial laboratory (Dairy One Forage Laboratory, Ithaca, NY, USA). The nutritive characteristics of the feed offered are presented in Table 1.

Three days prior to the measurement period, all cows were fitted with jaw movement recorders (RumiWatch, ITIN+HOCH GmbH, Liestal, Switzerland) to quantify eating behaviour. The halters remained on the cows for the entire measurement period and enabled the automatic measurement of time spent eating, ruminating and not chewing. The halters collected data via an inbuilt pressure sensor and a triaxial accelerometer.

Milk yield was recorded at each milking throughout the experiment using a DeLaval Alpro milk metering system (DeLaval International; Tumba, Sweden), and a sub-sample was collected for each cow using in-line milk meters (DeLaval International). Samples were analysed for fat, protein and lactose concentrations using an infrared milk analyser (Model 2000, Bentley Instruments, Chaska, MN, USA). Energy-corrected milk (ECM) yield was calculated using the following formula [15]:

ECM (kg/cow day^−1^) = milk yield (kg/cow day^−1^) × [376 × fat (%) + 209 × protein (%) + 948]/3138 (1)

At the commencement of the measurement period, capsules for measuring ruminal fluid pH (KB5; Kahne limited, Auckland, New Zealand) were calibrated and inserted per fistula into the rumen of each cow. The capsules remained in the cows until the end of the measurement period. A 750 g weight was attached to each capsule to ensure it remained on the bottom of the rumen. Ruminal fluid pH was logged every 5 min, and the data were automatically stored in the devices. Capsules were removed once a week for 8 h to recalibrate the pH devices, and a linear interpolation was used to correct for any drift in readings from individual boluses. Following the validation in standard pH buffers (4.01 and 7.01), all data were downloaded, and boluses were recalibrated before re-insertion.

Beginning on day 3 of the measurement period, seven ruminal fluid samples were collected per cow per feed, with the first sample collected immediately prior to feeding and a sample collected every hour thereafter. Samples were collected per fistula using a 100 mL plastic syringe connected to a copper pipe directly inserted into the rumen. Fluid was collected from four different sites within the rumen. A 50 mL sub-sample was immediately poured off and centrifuged (4 °C, 4000× *g*, 10 min), while the pH of the remainder was measured using a benchtop pH meter (Orion star A211; Thermo Fisher Scientific, Schwerzenbach, Switzerland). A 0.5 mL aliquot of supernatant was then transferred to a tube containing 4.5 mL of dilute acid (0.1 M HCl) for later analysis of the ammonia concentration. An additional 5 mL aliquot was dispensed into a tube for analysis of VFA and lactate concentrations. Both sub-samples were stored at −20 °C until analyses. Volatile fatty acid concentrations were determined by capillary gas chromatography (Agilent 6890 GC; Agilent Technologies, Santa Clara, CA, USA) using a flame ionisation detector, auto-sampler and auto-injector, and a wide bore capillary column (BP21 column, 12 m × 0.53 mm internal diameter (ID) and 0.5 μm film thickness; SGE International, Ringwood, Victoria, Australia) with a retention gap kit (including a 2 m × 0.53 mm ID guard column). Analyses were conducted following the methodology described by Packer et al. [16], with 4-methyl-valeric acid (1.58 mmol/L) used as the internal standard. Lactate analyses were conducted with a microplate reader (AMR-100, Allsheng Instruments, Hangzhou, China) using a D/L-lactate kit (K-DLATE; Megazyme, Bray, Ireland). Ammonia concentrations were determined by flow injection (Lachat Quik-Chem 8000; Lachat Instruments, Milwaukee, WI, USA) according to an alkaline phenol-based method (method 12-107-06-1-A; Lachat Instruments, Milwaukee, WI, USA) and analysed against standard ammonia solutions.

All data were analysed using Genstat for Windows (Genstat 18th edition, VSN International Ltd, Indore, India.). For all datasets, days were grouped according to diet, with days 1 and 2 categorised as forage only and day 3 categorised as forage and wheat. As day 4 only consisted of an a.m. period, it was not included in the overall analyses. Comparisons between forage groups hay (lucerne hay and perennial ryegrass hay) and fresh (perennial ryegrass cultivar Bealey and cultivar Base) as well as between forages within these groups, for all variables, were achieved by specifying contrasts on the factor for forage within the treatment structure employed in the ANCOVA. Daily yields (milk, ECM and composition yields) were calculated as the sum of p.m. and a.m. values. Daily milk composition (%) was calculated as the ratio of daily composition yield to milk yield. Milk production and intake data were subject to an analysis of variance (ANOVA) adjusted for data collected during the covariate period. The factorial treatment structure was forage by wheat, with a blocking structure of cow split for period (forage, wheat and forage) split for day.

The pH data from 2 intraruminal capsules were not able to be retrieved, one from a cow in the perennial ryegrass hay treatment and one from a cow in the lucerne hay treatment. Ruminal fluid pH data collected via the intraruminal capsules were summarised daily for each cow as daily mean, minimum, maximum, time under pH 6, area under pH 6 and rate of decline post-feeding. A day was considered from 07:00 h to 07:00 h. To calculate the rate of pH decline following each feeding, each daily set of pH data was also categorised into two ‘peak’ pH intervals and two ‘trough’ pH intervals. These intervals were derived visually from an average ruminal fluid pH (averaged over all cows, at each time) vs. time graph. The daily intervals were peak: from 03:00 to 09:00 h and 14:00 to 18:00 h, and trough: 09:00 to 14:00 h and 18:00 to 03:00 h. The maximum pH within each peak interval and the minimum pH within each trough interval were then identified and the slope (change in pH divided by change in time) was calculated. The data were then summarised as an average daily rate of decline in pH for each cow, the amount of pH decline and the duration of the decline. All summary data for ruminal fluid pH variables were subjected to an ANCOVA with a blocking structure of cow by period (forage, wheat and forage) split for day, with covariate as the corresponding variable measured in the covariate period. The factorial treatment structure was period by forage. Ruminal fluid fermentation profile data consisted of pre-feed and 6 h post-feed measurements for the morning and evening on each of day 2 and day 3. These were subjected to ANOVA with the factorial treatment structure of forage by period by sample (pre- or post-feeding) plus time of day (a.m. or p.m.), and a blocking structure of cow by period (i.e., day) split for time of day split for sample. Lactate data were log transformed prior to analysis. Eating behaviour data were analysed with an ANOVA using the treatment structure forage by wheat and the blocking structure cow by period split for day.

## 3. Results

### 3.1. Dry Matter Intake

Forage DMI varied with the type of forage (Table 2). When forage only was offered, cows offered perennial ryegrass hay consumed the least amount of forage (11.1 kg DM/cow day^−1^), while there was no difference between the other three treatment groups (15.1 kg DM/cow day^−1^). Cows in all treatments consumed all wheat that was offered and total DMI increased for all treatment groups on the day wheat was offered. Only lucerne hay-fed cows exhibited substitution effects, with the amount of forage consumed reducing following the consumption of wheat. This substitution effect resulted in an interaction between the effects of forage and wheat when comparing the herbage treatments to the hay treatments, such that the increase in total DMI when wheat was included was much greater for the herbage-fed cows.

### 3.2. Eating Behaviour

On a forage-only diet, cows fed lucerne hay spent more time eating than cows fed perennial ryegrass hay, but there was no difference in eating time between hay- and herbage-fed cows. On a forage-only diet, cows consuming hay spent, on average, an extra 269 min/day ruminating compared to cows fed herbage. For all treatments, the addition of wheat caused a change to the time spent eating. Cows fed either lucerne hay or herbage spent more time eating once wheat was included, while cows fed perennial ryegrass hay (PRG) hay reduced the time eating in response to the wheat. Time spent ruminating decreased for cows fed PRG hay and cultivar Bealey, once wheat was added, while there was no change to ruminating time for cows fed lucerne hay or cultivar Base.

### 3.3. Milk Yield and Composition

Mean yields of milk and ECM, and mean concentrations of milk fat, protein and lactose, for cows on the four dietary treatments, are presented in Table 3. An interaction between the effects of forage type and wheat occurred, resulting in an increase in the milk yield and ECM yield of herbage-fed cows when wheat was offered, while there was no change for hay-fed cows. With the addition of wheat to the diet, the milk yield of the cows fed perennial ryegrass cultivar Base increased, but this was not reflected in a difference in ECM yield. For the other three treatments, the inclusion of wheat in the diet did not affect milk yield or ECM. The only difference in milk composition was a higher lactose percentage from herbage-fed cows compared to those fed hay.

### 3.4. Ruminal Fluid pH and Fermentation Profile

**Figure 1:**
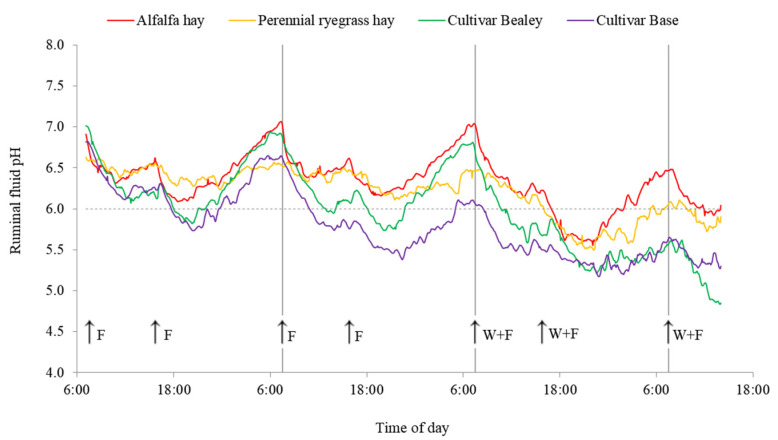
Changes in ruminal fluid pH over the 80-h measurement period for cows fed either lucerne hay, perennial ryegrass hay, perennial ryegrass herbage cultivar Bealey or perennial ryegrass herbage cultivar Base. Values are the raw means for treatments. Arrows indicate when feed was offered, F is a meal of forage only and WF is when wheat was fed followed by forage. The horizontal dashed line at pH 6.0 defines the ruminal fluid pH below which fibre digestion theoretically declines. The vertical lines indicate the beginning and end of each defined day.

Changes in ruminal fluid pH over the entire measurement period are presented in Figure 1. Ruminal fluid pH data for the morning of day 4 are presented in the figure but are not included in any of the analyses. Ruminal fluid pH characteristics on days 1 to 3 are presented in Table 4. Both mean and minimum ruminal fluid pH varied with forage type, being greatest for lucerne hay and perennial ryegrass hay, intermediate for Bealey and lowest for Base. Overall, for mean ruminal fluid pH, there was no interaction between the effects of forage type and wheat introduction, as the mean pH of all treatment groups declined similarly with the introduction of wheat. However, there was an interaction when herbage was compared to hay. The decline in mean ruminal fluid pH that occurred for the herbage treatment groups was much greater than that of the hay treatment groups (0.4 vs. 0.6 pH units). Minimum ruminal fluid pH also declined for all forages with the introduction of wheat, but no interaction effect occurred between forage and wheat. On average, the addition of wheat into the diet did not change the maximum pH of cows consuming hay but caused a reduction of 0.38 pH units for cows consuming herbage. The reduction was greatest for the Base treatment group (0.55 pH units).

The ruminal fluid of herbage-fed cows had a pH below 6.0 for a greater proportion of the day than the ruminal fluid of hay-fed cows; both on a forage-only diet and when wheat was included. On a forage-only diet, the ruminal fluid pH of cows consuming hay only briefly fell below 6.0 (0.8 h/cow per day). Herbage-fed cows had a ruminal fluid pH below 6.0 for a significantly longer period of time each day, particularly cows fed Base (11.2 h/cow per day). Following supplementation with wheat, the time ruminal fluid pH was below 6.0 increased for all treatments. For cows fed herbage, ruminal fluid pH was below 6.0 for almost the entire day (21.5 h/cow per day). For cows consuming perennial ryegrass hay, the duration of time below pH 6.0 increased from 0 to 12.9 h/cow per day, and for cows fed lucerne hay, the duration increased from 1.5 to 9.0 h/cow per day.

Forage type affected the concentration of VFA in the ruminal fluid (Table 5), with the greatest concentration in the herbage treatment groups, followed by the lucerne hay treatment group, and the lowest in the perennial ryegrass hay treatment group. The ruminal fluid mean concentration of acetate (expressed as a molar percentage of total VFA) was greater in cows fed hay compared to those fed herbage (68.2 and 60.7%, respectively), whereas the concentration of propionate was greater in the herbage-fed cows (18.8 and 21.2%, respectively). The concentration of butyrate was greatest in the herbage-fed cows, followed by perennial ryegrass hay, and lowest in the lucerne hay-fed cows (13.1, 10.5 and 9.1%, respectively). There was a main effect of wheat introduction, which led to increased concentrations of total VFA, propionate and butyrate, but a decreased concentration of acetate and acetate-to-propionate ratio. Adding wheat to the diet increased valerate concentrations for all treatments. However, the increase was twice as much for the perennial ryegrass hay and herbage treatments compared to the lucerne hay treatment (0.4 vs. 0.2%). Both before and after the inclusion of wheat, the concentration of valerate was much greater in the herbage treatments compared to the hay treatments. D/L-lactate concentrations (Table 5) were also affected by an interaction between forage and wheat. For cows fed herbage, D/L-lactate concentrations increased when wheat was added to the diet. For cows fed hay, however, D/L-lactate concentrations did not change with the inclusion of wheat. Ammonia N concentrations (Table 5) in herbage-fed cows were more than double the concentrations measured in hay-fed cows (125 and 260 mg/L) but were not impacted by wheat.

### 4. Discussion

The type of forage being consumed had significant effects on the ruminal fluid pH response to a wheat grain challenge. Compared with herbage, hay facilitated conditions in the rumen better able to cope with the accumulation of acid as a result of the sudden introduction and digestion of highly fermentable starch. Both with and without wheat in the diet, the daily mean and minimum ruminal fluid pH values were much greater for the cows consuming hays compared to those consuming the fresh forages. Furthermore, the ruminal fluid pH of cows fed fresh forages remained below 6.0 for a greater proportion of the day. The lower ruminal fluid pH from the herbage-fed cows was most likely due to greater VFA production rates [11]. Although VFA production rates were not measured, the lower NDF and higher ME of the herbage would suggest faster degradation rates [17], and this was further supported by higher concentrations of VFA measured in the herbage-fed cows, which has been associated with higher VFA production rates [18]. Saliva would have also played a major role in maintaining the ruminal fluid pH of the hay-fed cows. While intake is a driver of fermentation and hence acid production, saliva is the strongest buffer within the rumen [19], and saliva production is greatest during rumination [20,21]. Cows consuming hay were spending twice as long ruminating, driven by the greater NDF fraction [11].

The introduction of wheat into the diet dramatically increased the amount of time per day that ruminal fluid pH was below 6.0. The duration of time that pH remains below optimal is more influential on rumen function than the daily mean pH [22,23]. If pH falls below the 6.0 threshold only temporarily, the negative implications on fibre digestion are only small and transient. When low pH (<6.0) is sustained, however, the cellulolytic bacterial populations can be compromised [22]. Low ruminal fluid pH not only reduces fibre digestion [24] but can also limit energy intake and protein absorption due to the negative impacts on ruminal fluid motility, microbial yield and appetite [22,25]. If ruminal fluid pH is reduced to levels below 6.0 and remains there for extended periods, severe health problems can arise such as liver abscesses, laminitis, digestive tract tissue damage and, in extreme cases, death [26,27,28].

Following wheat supplementation, the herbage-fed cows had ruminal fluid pH values below 6.0 for almost the entire day. This is clear evidence that gradual adaptation strategies must be used to introduce large amounts of wheat when cows are consuming highly digestible herbage. The ruminal fluid of lucerne hay-fed cows proved most resistant to the supplementation of wheat, exhibiting the smallest increase in time below pH 6.0. Despite having no prior wheat adaptation, the time below pH 6.0 was almost half that described in previous work when cows were grazing fresh Persian clover, at an average amount of 19 kg DMI/cow day^−1^, and adapted over 12 days to wheat fed at 3 kg DM/cow day^−1^ [10]. Comparatively, these results demonstrate how varied the adaptation process can be with different forages. However, it is possible that time below pH 6.0 would have increased for the lucerne hay-fed cows with continued wheat supplementation. It is also possible the results may have differed if the pasture was grazed instead of harvested for feeding. High allowances of grazed pasture would have allowed for greater selection through more opportunity, possibly resulting in higher intakes and different nutritive profiles. Previous research has shown that higher allowances lead to increased DMI and increased nutrient intake of CP and sometimes ME [29,30].

Although the maximum pH values reported for Bealey and Base ryegrass cultivars in a forage and wheat diet were both above 6.0 (6.84 and 6.24, respectively), these values were recorded immediately after the morning feed was offered. From that time point onwards, ruminal fluid pH declined and, over the final 31 h, remained at levels known to compromise NDF digestion [31]. This downward trend continued further during the observations on day 4 (Table 6) when the maximum pH reached was 5.65 for Bealey and 5.81 for Base; again, these values were observed at the start of the day, followed by a downward trend. This was likely driven by the lower NDF concentrations and higher ME concentration of the pastures, resulting in a faster rumen passage rate and very little feed in the rumen prior to wheat consumption. This, combined with reduced rumination times, meant there were relatively less buffers available to resist further declines in pH with the fermentation of wheat. The ruminal fluid pH of cows in the herbage treatment groups showed very little ability to recover. It is possible that the sustained low pH levels reduced cellulolytic microflora [6], including protozoa that help maintain a higher ruminal pH by engulfing starch granules [32]. Hence, the low pH was further exacerbated. The ruminal fluid pH of cows in both the lucerne hay and perennial ryegrass hay treatment groups recovered to levels above 6.0 at the beginning of day 4, values similar to those reported on a forage-only diet.

On day 2, during the forage-only period, the ruminal fluid pH of the Base treatment group was below pH 6.0 for almost the entire day, indicating that even without wheat in the diet, fibre digestion may have been impaired. Ruminal fluid with a pH this low on a diet of solely perennial ryegrass pasture has previously been reported by Williams et al. [12,33]. The lower ruminal fluid pH on day 2 compared to day 1 for the Base treatment group is likely due to the greater DMI on day 2. The cows consumed about 4 kg DM/cow more on day 2 compared to day 1 (12.6 v 16.8), which resulted in a lower ruminal fluid pH, a result previously reported in both stall-fed and grazing dairy cows [33,34]. The already low ruminal fluid pH on the herbage-only diet indicated SARA was already prevalent in these cows prior to wheat supplementation.

Unlike the other three treatment groups, the average 24 h ruminal fluid pH pattern exhibited by cows fed perennial ryegrass hay only was not a W-shaped pattern, as is typical when cows are fed twice daily [35,36]. Rather, the ruminal fluid pH showed very little variation, varying by 0.55 pH units compared to 1.05 pH units for lucerne hay. This was likely due to the lower and slower intakes by the cows fed perennial ryegrass hay. While reduced variability benefits fibre digestion at low pH levels [37], the mean pH of lucerne-fed cows was relatively high, remaining above pH 6.0 both before and after wheat supplementation. This indicates that the reduced variability would have provided no benefit for perennial ryegrass hay-fed cows over those fed lucerne hay. For the herbage treatments, however, the large variability paired with the low mean pH in the forage–wheat diet likely posed significant threats to fibre digestion.

There were greater proportions of propionate and butyrate in the ruminal fluid of herbage-fed cows, which is consistent with the lower NDF concentration of the feed, while the greater proportion of valerate was likely driven by the higher CP concentration of the herbage [38,39]. The change in the VFA proportion with the addition of wheat was consistent across treatments. The proportion of acetate declined, while the proportions of propionic, butyrate and valerate all increased, reflecting the reduced proportion of VFA produced from NDF digestion and the greater contribution of starch digestion [38]. The higher concentration of valerate in cows with SARA is supported by the results of Bramley et al. [40].

Observations made on day 4 (Table 6) highlighted the degree to which the herbage-fed cows were struggling to cope with the grain challenge, and symptoms indicated acute acidosis [41]. Rumination during the 7 h observation period had all but completely stopped for both Bealey and Base treatment groups. Cows in the Bealey treatment group appeared most compromised, exhibiting a minimum ruminal fluid pH of 4.78, and D/L-lactate concentrations were eight times greater than the previous day, contributing significantly to the total acid load, which is responsible for acidosis [42]. The order of the feeding, wheat before forage, may have played an important role in dictating pH patterns. Hay-fed cows would have returned for the following feed with forage remaining in the rumen, allowing for buffering against the acids produced immediately by wheat fermentation. Cows consuming fresh herbage, however, were likely consuming wheat with a near empty rumen, resulting in dramatic declines in ruminal pH.

The benefits of mitigating the impacts of dietary adaptation are extensive. Successful adaptation to a high-concentrate diet improves the welfare of dairy cows by avoiding SARA and acute ruminal acidosis, both of which are concerns for the Victorian dairy industry [40,43]. Furthermore, if the time required for successful adaptation to a high-concentrate diet can be reduced, as indicated by the lucerne hay treatment within this study, total ME intake can be increased more rapidly, creating potential for increased milk production [13]. The results of the current experiment indicate that there should be a focus on forage type when deciding on appropriate concentrate introduction strategies.

## 5. Conclusions

The ruminal environment of cows fed hay had an ability to resist significant declines in ruminal fluid pH that are typically associated with rapid concentrate adaptation. This contrasted with cows fed herbage, which exhibited symptoms associated with SARA, including more than 20 h of the day with a ruminal fluid pH below 6.0. Overall, these findings highlight a potential to more rapidly introduce large amounts of wheat grain to forage-fed cows when high-quality hay is the basal forage.

## Figures and Tables

**Table 1:** Nutritive characteristics of feed offered during the experimental period ^1^ (CP, crude protein; ADF, acid detergent fibre; aNDF, neutral detergent fibre; NFC, non-fibre carbohydrates; CF, crude fat (ether extract); ME, metabolisable energy).

	CP	ADF	aNDF	Lignin	NFC	Starch	CF	Ash	ME ^2^
Lucerne hay	14	47	55	10	21	0.9	2.6	8.1	8.9
Ryegrass hay	10	40	60	8	23	1.4	1.9	5.5	8.9
Ryegrass (Bealey) herbage	28	40	46	10	10	1.3	5.8	10.4	10.2
Ryegrass (Base) herbage	29	42	48	12	8	0.9	6.1	9.6	10
Wheat	14	5	11	2	71	58.7	2.2	1.8	14.4

^1^ All values are % of DM unless otherwise indicated; ^2^ MJ/kg DM.

**Table 2:** Influence of forage type and the addition of wheat to the diet on feed intake (kg DM/cow day^−1^) and eating behaviour (min/cow day^−1^) ^1^.

		Feed Intake			Eating Behaviour		
Forage	Diet	Forage	Wheat	Total	Eating	Ruminating	Not Chewing
Lucerne hay	Forage only	16.5	0	16.5	393	484	548
	Forage + wheat	13.7	7.5	21.3	451	478	498
Ryegrass hay	Forage only	11.1	0	11.1	359	584	488
	Forage + wheat	9.7	7.5	17.3	284	454	690
Ryegrass (Bealey) herbage	Forage only	13.9	0	13.9	355	295	782
	Forage + wheat	14.3	7.5	21.8	418	246	764
Ryegrass (Base) herbage	Forage only	14.8	0	14.8	368	236	827
	Forage + wheat	15.3	7.5	22.8	446	237	745
	SED	0.86		0.86	36.5	21.9	19.4
*p* value	Forage	<0.001		<0.001	0.185	<0.001	<0.001
	Hay v herbage	0.031		0.031	0.643	<0.001	<0.001
	Ryegrass v lucerne	<0.001		<0.001	0.042	0.169	0.651
	Bealey v Base	0.239		0.239	0.602	0.312	0.649
	Wheat	0.083		<0.001	0.002	0.034	0.532
	Forage × Wheat	0.060		0.060	<0.001	0.115	0.001
	Hay v herbage	0.013		0.013	<0.001	0.275	0.008
	Ryegrass v lucerne	0.291		0.291	<0.001	0.042	<0.001
	Bealey v Base	0.930		0.930	0.503	0.382	0.279

^1^ Values are treatment means from days 1 and 2 (forage only), or day 3 (forage and wheat).

**Table 3:** Influence of forage type and the addition of wheat to the diet on milk yield (kg/cow per d), energy-corrected milk (ECM) yield (kg/cow per d) and milk composition (%) ^1,2^.

Forage	Diet	Milk Yield	ECM	Fat	Protein	Lactose
Lucerne hay	Forage only	15.6	16.9	5.1	3.2	4.6
	Forage + wheat	15.8	15.9	4.5	3.3	4.6
Ryegrass hay	Forage only	7.7	8.9	5.2	3.6	4.2
	Forage + wheat	8.1	8.7	4.8	3.5	4.3
Ryegrass (Bealey) herbage	Forage only	17.9	20.2	4.9	3.7	4.9
	Forage + wheat	19.2	21.5	4.9	3.5	4.9
Ryegrass (Base) herbage	Forage only	16.9	18.8	4.9	3.4	4.7
	Forage + wheat	21.2	22.4	4.5	3.4	4.7
	SED	0.92	1.27	0.38	0.14	0.14
*p* value	Forage	0.002	0.001	0.950	0.277	0.072
	Hay v herbage	0.001	<0.001	0.742	0.431	0.028
	Ryegrass v lucerne	0.004	0.006	0.746	0.167	0.153
	Bealey v Base	0.996	0.788	0.740	0.241	0.519
	Wheat	0.005	0.164	0.093	0.326	0.426
	Forage × Wheat	0.029	0.105	0.735	0.810	0.911
	Hay v herbage	0.019	0.034	0.466	0.745	0.622
	Ryegrass v lucerne	0.850	0.636	0.656	0.468	0.687
	Bealey v Base	0.040	0.218	0.487	0.598	0.760

^1^ All values are covariate adjusted; ^2^ values are treatment means from days 1 and 2 (forage only), or day 3 (forage and wheat).

**Table 4:** Influence of forage type and the addition of wheat to the diet on mean ruminal fluid pH characteristics ^1,2^.

Forage	Diet	Mean	Minimum	Maximum	Time under pH 6 ^3^	Area under pH 6 ^4^
Lucerne hay	Forage only	6.43	6.05	7.10	1.5	0.3
	Forage + wheat	6.08	5.47	7.05	9.0	2.7
Ryegrass hay	Forage only	6.43	6.11	6.66	0.0	0.1
	Forage + wheat	5.97	5.37	6.57	12.9	4.2
Ryegrass (Bealey) herbage	Forage only	6.26	5.76	7.04	5.8	1.0
	Forage + wheat	5.63	5.15	6.84	20.6	9.7
Ryegrass (Base) herbage	Forage only	6.07	5.55	6.79	11.2	2.9
	Forage + wheat	5.53	5.06	6.24	22.3	12.0
	SED	0.084	0.130	0.095	1.79	1.03
*p* value	Forage	<0.001	<0.001	<0.001	<0.001	<0.001
	Hay v herbage	<0.001	<0.001	0.279	<0.001	<0.001
	Ryegrass v lucerne	0.610	0.865	<0.001	0.839	0.610
	Bealey v Base	0.039	0.029	0.001	0.017	0.011
	Wheat	<0.001	<0.001	<0.001	<0.001	<0.001
	Forage × Wheat	0.062	0.651	<0.001	0.025	0.002
	Hay v herbage	0.018	0.424	<0.001	0.078	<0.001
	Ryegrass v lucerne	0.346	0.466	0.698	0.028	0.270
	Bealey v Base	0.294	0.530	<0.001	0.070	0.761

^1^ Summary of ruminal fluid pH characteristics days 1 and 2 (forage only), and day 3 (forage and wheat); ^2^ values are covariate adjusted; ^3^ mean time per day during which ruminal fluid pH was below 6.0 (h); ^4^ area of the pH vs. time of day curve below pH 6.0 (pH × h).

**Table 5:** Influence of forage type and the addition of wheat to the diet on mean concentrations in ruminal fluid of total volatile fatty acids (Total) and acetic acid (Ace), propionic acid (Pro), butryric acid (But) and valeric acid (Val; all in mmol/L), as well as ammonia N (Am N; mg/L) and D/L-lactate (Lac; mmol/L) ^1^.

Forage	Diet	Total	Ace	Pro	But	Val	Ace:Pro	Am N	Lac ^2^
Lucerne hay	Forage only	122	71.3	17.0	8.2	1.2	4.2	146	0.040
	Forage + wheat	123	67.2	19.0	10.0	1.4	3.6	159	0.015
Ryegrass hay	Forage only	93	69.9	18.2	9.6	0.9	3.9	51	0.055
	Forage + wheat	111	64.4	21.0	11.4	1.3	3.1	145	0.022
Ryegrass (Bealey) herbage	Forage only	141	62.3	20.8	12.2	1.4	3.0	208	0.520
	Forage + wheat	155	58.5	23.4	12.9	1.8	2.5	292	0.632
Ryegrass (Base) herbage	Forage only	144	63.1	18.9	13.1	1.4	3.4	244	0.038
	Forage + wheat	162	58.9	21.5	14.1	1.8	2.8	293	0.371
	SED	7.9	1.19	1.12	0.72	0.09	0.22	62.0	0.7410
*p* value	Forage	<0.001	<0.001	0.013	<0.001	<0.001	<0.001	0.008	0.069
	Hay v herbage	<0.001	<0.001	0.007	<0.001	<0.001	<0.001	0.001	0.043
	Ryegrass v lucerne	0.002	0.067	0.146	0.016	0.021	0.055	0.248	0.068
	Bealey v Base	0.311	0.556	0.088	0.063	0.752	0.129	0.689	0.918
	Wheat	0.011	<0.001	<0.001	0.004	<0.001	<0.001	0.068	0.596
	Forage × Wheat	0.475	0.472	0.824	0.686	0.040	0.578	0.769	0.014
	Hay v herbage	0.451	0.339	0.763	0.252	0.086	0.297	0.834	0.005
	Ryegrass v lucerne	0.189	0.233	0.387	0.944	0.016	0.500	0.356	0.072
	Bealey v Base	0.733	0.738	0.976	0.812	0.872	0.548	0.686	0.558

^1^ Data are mean values from samples taken 6 h after feed was offered in both the morning and evening on days 1 and 2 (forage only), and day 3 (forage and wheat); ^2^ means were log transformed for analysis. Values presented are raw means, while SED refers to log-transformed values.

**Table 6:** Means of feed intake, eating behaviours, ruminal fluid pH and ruminal fluid composition of cows receiving each treatment as observed on day 4 of wheat inclusion ^1^.

Item	Lucerne Hay	Ryegrass Hay	Ryegrass (Bealey) Herbage	Ryegrass (Base) Herbage
Feed intake (kg DM/cow)				
Forage	4.7	1.8	3.7	3.2
Wheat	4.0	3.0	2.9	2.9
Total	8.7	4.8	6.6	6.0
Eating behaviour (min/cow)				
Eating	164	102	141	130
Ruminating	102	86	3	6
Not chewing	149	227	274	282
Ruminal fluid pH				
Mean	6.14	5.93	5.26	5.44
Minimum	5.91	5.71	4.78	5.18
Maximum	6.55	6.26	5.65	5.81
Ruminal fluid composition ^2^				
Total VFA (mmol/L)	130	124	184	170
Acetate (molar %)	65.7	61.7	59.6	58.5
Propionate (molar %)	20.2	19.4	20.0	18.8
Butyrate (molar %)	10.1	15.1	16.0	17.1
Valerate (molar %)	1.4	1.2	1.4	1.8
Acetate: Propionate	3.3	3.2	3.0	3.1
Ammonia N (mg/L)	96	12	377	340
D/L-Lactate (mmol/L)	0.028	0.013	5.377	1.034

^1^ The observation period was from 07:00 to 14:00 h. Cows had received wheat and forage in the morning; ^2^ as sampled 6 h post-feed.

## Data Availability

Not applicable.
